# Context‐Specific Metabolic Alterations in HPRT1 Knockout Cells Within a 3D Culture System

**DOI:** 10.1002/cam4.71452

**Published:** 2025-12-09

**Authors:** Sho Tabata, Ichiro Fujimoto, Tomoyoshi Soga, Hideki Makinoshima

**Affiliations:** ^1^ Tsuruoka Metabolomics Laboratory National Cancer Center Tsuruoka Japan; ^2^ Shonai Regional Industry Promotion Center Tsuruoka Japan; ^3^ Division of Translational Informatics, Exploratory Oncology Research and Clinical Trial Center National Cancer Center Kashiwa Japan; ^4^ Koken Research Institute, Koken Co., Ltd. Tsuruoka Yamagata Japan; ^5^ Institute for Advanced Biosciences Keio University, Tsuruoka Tsuruoka Japan

**Keywords:** 3D culture model, CARNS1, HPRT1, metabolome, purine metabolism, SCLC

## Abstract

**Background:**

Cancer cells reprogram their metabolism to sustain energy production and biosynthesis for malignant proliferation; however, their metabolic phenotypes vary significantly across different growth environments, creating discrepancies between in vitro and in vivo findings. These inconsistencies pose challenges for translating metabolic research into clinical applications. The emergence of 3D culture models as in vitro systems that accurately mimic the in vivo environment can mitigate these challenges by providing conditions that reflect physiological architecture and metabolic interactions. Therefore, we investigated the impact of the purine metabolism enzyme hypoxanthine–guanine phosphoribosyltransferase 1 (HPRT1) on the proliferation and metabolism of SCLC cells using 2D and 3D culture models, with the goal of identifying context‐specific metabolic regulation not captured in conventional 2D cultures.

**Methods:**

We evaluated cell proliferation and performed metabolomic profiling of HPRT1‐knockout (KO) SCLC cells grown in 2D cultures, two 3D culture systems, and mouse xenograft models. Metabolomic profiling was performed using CE‐TOFMS, followed by PCA and pathway analysis. The expression of β‐alanine (β‐Ala) metabolism‐related genes, including carnosine synthase 1 (CARNS1), was assessed by RNA‐seq and RT‐PCR. CARNS1 expression was further evaluated in publicly available lung cancer datasets, including both cell line and clinical tumor cohorts, to determine its association with patient prognosis and its correlation with HPRT1 expression.

**Results:**

The knockout of HPRT1 significantly reduced cell proliferation in 3D cultures and in vivo but had a minimal impact in 2D cultures. Comprehensive metabolomic analyses of HPRT1‐KO cells revealed extensive alterations in amino acid and purine metabolism both in vitro and in vivo. Notably, the effect of HPRT1 KO on β‐Ala metabolism differed between 2D and 3D cultures. In 3D cultures, HPRT1 KO led to increased expression of the endogenous antitumor metabolite carnosine and its biosynthetic enzyme CARNS1 within the β‐Ala metabolic pathway. Furthermore, analysis of clinical databases showed that high CARNS1 expression correlated with improved prognosis in patients with lung cancer and negatively correlated with HPRT1 expression in tumor tissues.

**Conclusion:**

This study highlights the potential of 3D culture systems to elucidate context‐specific mechanisms of metabolic regulation, such as the suppressive effect of HPRT1 on carnosine production. Our findings demonstrate that metabolic phenotypes observed in 2D cultures may not fully capture the complexity of in vivo metabolism, whereas 3D models can reveal regulatory pathways that are otherwise overlooked, including context‐dependent regulation of carnosine metabolism by HPRT1.

## Graphical abstract

Cancer metabolism studies in 2D culture models often overlook the in vivo complexity. We found that HPRT1 knockout suppressed cell proliferation in 3D and in vivo, but had minimal effect in 2D. Metabolomic analysis also revealed that HPRT1 knockout increased carnosine production via the β‐alanine pathway in 3D cultures.

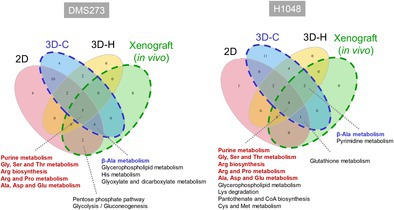

**Abbreviations**
β‐Ala: β‐Alanine
CARNS1: Carnosine synthase 1
CE‐TOFMS: capillary electrophoresis time‐of‐flight mass spectrometry
HPRT1: hypoxanthine–guanine phosphoribosyltransferase 1
MPA: metabolic pathway analysis
PCA: principal component analysis
SCLC: small cell lung cancer

## 1 Introduction

Cancer cells exhibit unique metabolic adaptations to meet the heightened energy and biosynthetic demands of malignant proliferation. The enhancement of aerobic glycolysis, also known as the Warburg effect, is particularly well‐documented [1, 2]. Cancer cell metabolism is profoundly influenced by the surrounding growth environment, frequently leading to substantial metabolic differences between in vitro and in vivo conditions [3, 4]. Recent studies have emphasized the importance of the tumor microenvironment in modulating these metabolic behaviors [5, 6, 7]. These discrepancies present considerable challenges for cancer metabolism research.

For instance, lung cancer cells predominantly use glutamine to fuel the tricarboxylic acid (TCA) cycle in vitro, whereas glucose is the preferred substrate in vivo [8, 9, 10]. Furthermore, the knockout (KO) of enzymes such as pyruvate dehydrogenase and pyruvate carboxylase in lung cancer cells does not impact proliferation under 2D culture conditions but markedly suppresses tumor formation in mouse xenograft models [10]. In contrast, mitochondrial glutamate‐oxalate transaminase 2 KO inhibits the growth of pancreatic ductal adenocarcinoma in vitro but does not impact tumor growth in vivo [11]. This divergence between in vitro and in vivo functions is also observed in several metabolic enzymes, such as *N*‐acetylglucosamine kinase [12], pyruvate dehydrogenase kinase 4 [13], and genes involved in heme synthesis [14].

Previously, we investigated the role of hypoxanthine–guanine phosphoribosyltransferase 1 (HPRT1), an enzyme implicated in the purine salvage pathway, in small cell lung cancer (SCLC) [15]. HPRT1 contributes to the malignant proliferation of SCLC in vivo and mediates resistance to antifolate drugs inhibiting de novo purine synthesis. Moreover, HPRT1 KO had a minimal effect on cell proliferation under in vitro 2D culture conditions but significantly suppressed tumor formation in mouse xenograft models. To further elucidate the metabolic function of HPRT1 beyond its role in drug resistance, in this study, we explored its impact on tumor metabolism using 2D, 3D, and in vivo models.

3D culture systems that accurately mimic in vivo environments present a promising alternative to animal experiments [16]. These systems address the limitations of traditional 2D cultures. However, the metabolic changes and phenotypic differences associated with 2D versus 3D cultures remain inadequately characterized.

In this study, we investigated the growth and metabolome of HPRT1‐KO SCLC cells using 2D and 3D culture models, along with a mouse xenograft model. Our findings highlight the utility of 3D culture systems in cancer metabolism research and provide insights into their significance for understanding tumor biology.

## 2 Methods

### 2.1 Cell Culture

The SCLC cell lines DMS273 (European Collection of Authenticated Cell Culture, Salisbury, UK) and H1048 (American Type Culture Collection, Manassas, VA, USA) were used. All cells were grown in RPMI 1640 medium (Nacalai Tesque, Kyoto, Japan) supplemented with 10% (v/v) FBS (Biowest, Nuaillé, France) and incubated at 37°C in a humidified atmosphere with 5% CO_2_. All cells were annually tested for mycoplasma using the BioMycoX Mycoplasma PCR Detection Kit (CellSafe, Seoul, Korea). 3D collagen (3D‐C) and 3D honeycomb cultures (3D‐H) were conducted according to the manufacturer's instructions using 3D Ready Atelocollagen DMEM‐HG1 (KOKEN CO. LTD, Tokyo, Japan; 3D‐HG01) and 3D Honeycomb Boosted (KOKEN CO. LTD; 3D‐HCB), respectively. In the 3D cultures, the medium was replaced with fresh medium every 2 days. In metabolic analyses, the culture medium was replaced with fresh medium 24 h before sample collection.

### 2.2 Establishment of HPRT1‐Knockout SCLC Cells

DMS273 and H1048 cells with HPRT1 KO were established, as previously described [15]. HPRT CRISPR/Cas9 KO plasmids were purchased from Santa Cruz Biotechnology (sc‐417,332; Santa Cruz, CA, USA). The sgRNA sequences targeting the HPRT1 exon were obtained from three predesigned gRNAs: gRNA#A, 5′‐GTTATGGCGACCCGCAGCCC‐3′; gRNA#B, 5′‐CTGTCCATAATTAGTCCATG‐3′; and gRNA#C, 5′‐TCTTGCTCGAGATGTGATGA‐3′.

### 2.3 HPRT1 Expression via Retrovirus Transduction

*HPRT1* transduction was performed as previously described [15], using the retrovirus packaging cell line Platinum‐E and retroviral vector pMXs (Funakoshi, Japan) [17]. Plasmid was constructed using a retrovirus system according to the manufacturer's instructions, and human *HPRT1* was inserted. Transfection was performed using Lipofectamine 2000 reagent (Thermo Fisher Scientific, Waltham, MA, USA), according to the manufacturer's instructions.

### 2.4 RNA Isolation and Real‐Time PCR Analysis

RNA was isolated from cells using NucleoSpin RNA Plus (Macherey‐Nagel, Duren, Germany). Next, cDNA synthesis was performed using a SuperScript VILO cDNA Synthesis Kit (Thermo Fisher Scientific), followed by quantitative PCR using a TB Green Premix Ex Taq (TaKaRa Bio, Japan) with a StepOne Plus Real‐Time PCR system (Applied Biosystems, Foster City, CA, USA), according to the manufacturer's instructions. The ΔΔCq method was used to quantify gene expression, with GAPDH as an internal reference [18]. All experiments were performed in triplicate. The primers used for real‐time PCR are listed in Table S1.

### 2.5 Western Blotting

Immunoblot analysis was performed, as described previously [15]. Primary antibodies used included GAPDH (1:4000; Proteintech, MA, USA; 10494‐1‐AP) and HPRT1 (1:10,000; Abcam, Cambridge, MA, USA; ab109021). Horseradish peroxidase‐conjugated secondary antibodies (GE Healthcare, IL, USA) were used.

### 2.6 Metabolomic Analysis Using Capillary Electrophoresis Time‐of‐Flight Mass Spectrometry (CE‐TOFMS)

Intracellular metabolites were quantified using CE‐TOFMS (Agilent Technologies, Palo Alto, CA, USA), as described previously [19, 20, 21]. Metabolites were identified by matching their *m*/*z* values and migration times to those of standard compounds. Quantitative metabolite levels were corrected for the total DNA amount of each sample.

For data interpretation, MetaboAnalyst 6.0 software (http://www.metaboanalyst.ca) was used for principal component (PCA), metabolite pathway (MPA), and volcano plot analyses. This software was selected owing to its robust statistical algorithms, intuitive interface, and comprehensive pathway libraries, which ensure reliable data interpretation and biological contextualization. In the MPA (Figure 3), we identified pathways enriched with metabolites that were significantly altered by HPRT1 KO (FDR < 0.05) in 2D/3D culture or mouse xenograft models. Venn diagrams (Figure 5) were constructed using the pathways annotated with two or more metabolites significantly altered in the MPA.

### 2.7 Metabolomic Analysis of Xenografted Mouse Tumors

Animal experiments were conducted, as described previously [15]. All experimental nude mice were handled in accordance with the institutional guidelines established by the Animal Care Committee of the National Cancer Center. All the animal experiments were approved by the same committee (Approval number: #17502 and #21502). *HPRT1*‐KO cells were injected into the subcutaneous tissues of 8‐week‐old female nude mice (CLEA, Tokyo, Japan). After tumor formation, the tissues were harvested, immediately frozen in liquid nitrogen, and stored at −80°C until use. Metabolites were extracted and quantified using CE‐TOFMS, as described previously [22]. The metabolomic analysis of DMS273‐derived tumors constituted a reanalysis of data obtained from a previous study [15], whereas that of H1048‐derived tumors was carried out in the present study.

### 2.8 RNA‐Seq Analysis

Total RNA was isolated using NucleoSpin RNA Plus (Macherey‐Nagel). The sequencing library construction and RNA‐seq were performed by Macrogen Japan Corp. (Kyoto, Japan). Libraries were prepared using the TruSeq stranded mRNA Library kit (Illumina, San Diego, CA, USA) and sequenced on the NovaSeqX sequencer (Illumina) with 151‐bp paired‐end reads. Adaptor sequences and low‐quality bases were removed using Cutadapt (v4.9). The filtered reads were mapped onto the *Homo sapiens* reference genome (gs://genomics‐public‐data/resources/broad/hg38/v0/Homo_sapiens_assembly38.fasta) and the annotation file (GCF_000001405.40_GRCh38.p14_genomic.gtf) using STAR (v2.7.11a). Gene expression levels were quantified as transcripts per million using RSEM (v1.3.3). The PCA score plot for β‐Ala metabolism genes was depicted using MetaboAnalyst 6.0 software.

### 2.9 Correlation Analysis Between *CARNS1* and *HPRT1* mRNA Expression in Lung Cancer Cells and Tissues

CARNS1 and HPRT1 mRNA expression (RNA‐seq data) for 192 and 1129 lung cancer cell lines (Cancer Cell Line Encyclopedia, CCLE) [23, 24] and tissues (The Cancer Genome Atlas, TCGA) [25] were obtained from the CellMinerCDB (https://discover.nci.nih.gov/rsconnect/cellminercdb) and UCSC Xena (https://xena.ucsc.edu), respectively. The data were then visualized using scatter plots. Pearson's correlations and *p*‐values were calculated using the GraphPad Prism software (GraphPad Software Inc., La Jolla, CA, USA).

### 2.10 Kaplan–Meier Survival Analysis

Survival variance based on gene expression was determined using the Kaplan–Meier Plotter for lung cancer (https://kmplot.com/analysis/index.php?p=service&cancer=lung) [26]. The gene symbol “CARNS1 (228984_at)” was entered, and the auto checkbox was selected to determine the optimal cutoff value for *CARNS1* mRNA expression. Patients were then classified into high and low expression categories. Subsequently, univariate Cox regression analysis was conducted to compute the hazard ratio (accompanied by a 95% confidence interval) and *p*‐values. Four Gene Expression Omnibus datasets (GSE19188, GSE30219, GSE31210, and GSE37745) were utilized to assess the relationship between *CARNS1* expression and outcomes for patients with lung cancer.

### 2.11 Statistical Analysis

Significant differences between groups were determined using unpaired two‐tailed student's *t* test. All statistical analyses were performed using GraphPad Prism v8.0 software (GraphPad Software Inc.). Data are expressed as the mean ± standard deviation (SD). Statistical significance was set at *p* < 0.05.

## 3 Results

### 3.1 Metabolic Changes Under 3D Culture Conditions

**FIGURE 1:**
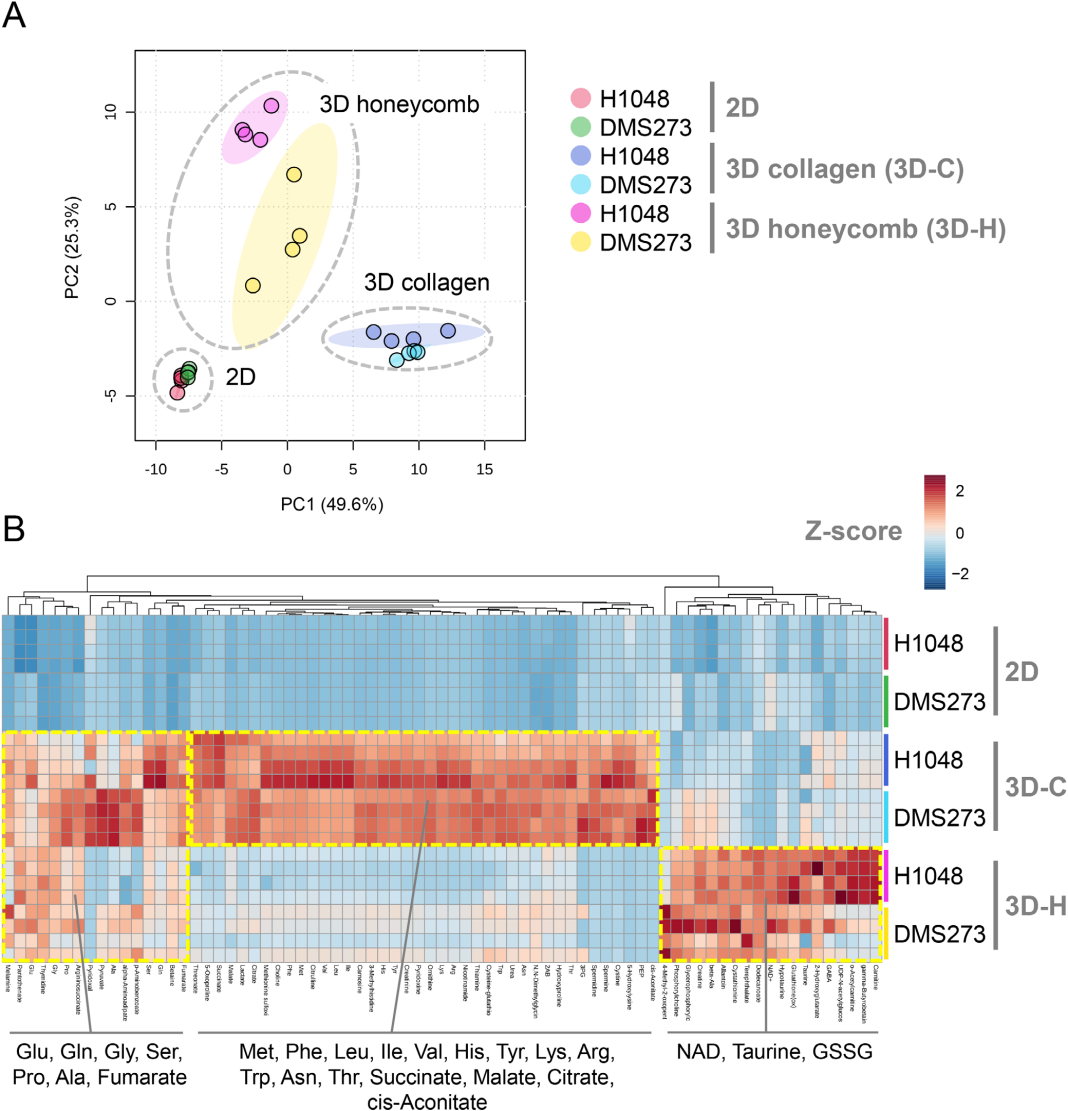
Metabolomic analysis of SCLC cells in 2D and 3D culture models. (A) Principal component analysis (PCA) of the intracellular metabolites in DMS273 and H1048 cells under 2D, 3D collagen (3D‐C), and 3D honeycomb (3D‐H) culture conditions. Metabolomic analysis was performed using CE‐TOFMS; *N* = 4. (B) Levels of intracellular metabolites in DMS273 and H1048 under 2D, 3D‐C, and 3D‐H culture conditions. The heatmap shows the *Z*‐score of relative metabolite expression for each sample.

We performed metabolomic analyses of SCLC cells grown in conventional 2D, 3D collagen, and 3D honeycomb cultures to investigate the metabolic alterations induced by 3D culture. The 3D collagen culture, which is widely used in 3D cell culture studies, grows cells in a collagen gel and provides a solid gel scaffold. In contrast, the 3D honeycomb culture method allows cells to grow in collagen with a honeycomb structure, where the cells exist either in a semi‐floating 3D state or adhere to the walls. DMS273 and H1048 cells were cultured in each culture condition, and their metabolomic profiles were analyzed using CE‐TOFMS (Figure 1 and Table S2). PCA score plots revealed distinct intracellular metabolic profiles among the three culture conditions (Figure 1A). Many amino acids and intermediates of the TCA cycle showed an increasing trend under 3D culture conditions (Figure 1B). Furthermore, differences in metabolic profiles were observed between the two 3D culture methods. Specifically, the levels of redox‐related metabolites such as NAD, taurine, and oxidized glutathione (GSSG) increased in the 3D honeycomb culture (Figure 1B). These results indicate that alterations in the metabolic state of cells depend on the culture model, suggesting that the function of metabolic genes is highly context‐dependent.

### 3.2 Proliferation of HPRT1‐KO Cells Under 3D Culture Conditions

**FIGURE 2:**
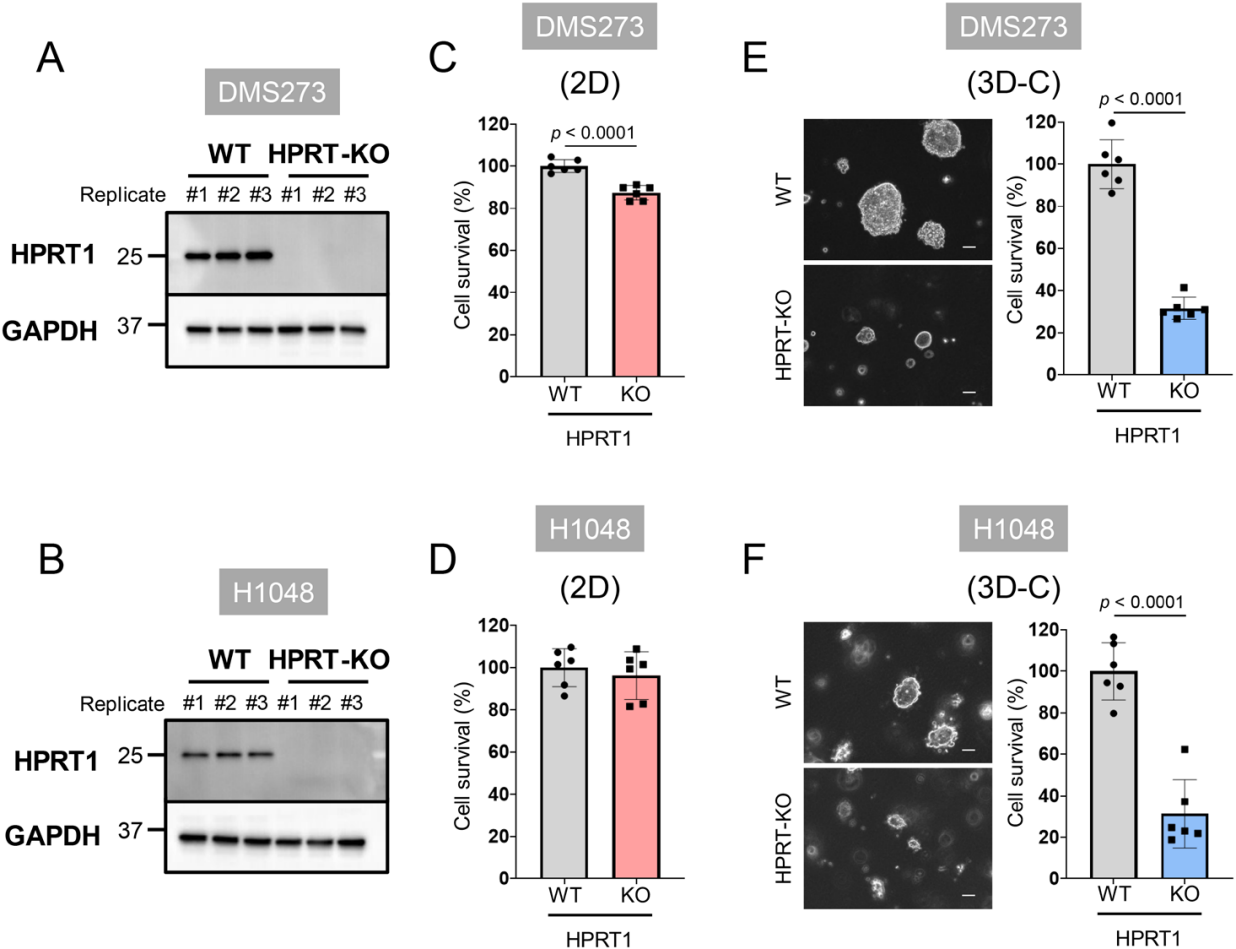
Growth of HPRT1‐KO SCLC cells under 2D and 3D culture conditions. (A, B) HPRT1 expression in the HPRT1‐KO DMS273 (A) and H1048 (B) cells. *HPRT1* was depleted using the CRISPR/Cas9 system. Protein expression levels were measured using western blotting. GAPDH was the loading control. *N* = 3. (C, D) The growth of HPRT1‐KO cells in the 2D culture model. DMS273 (C) and H1048 (D) cells were cultured for 3 days. Cell viability was evaluated using the WST‐8 method; *N* = 6. (E, F) The growth of HPRT1‐KO cells in the 3D collagen culture model. DMS273 (E) and H1048 (F) cells were cultured for 10 days. Right: Representative images. Scale, 50 μm. Left: Bar plots showing cell viability. *N* = 6. Data are expressed as the mean ± SD. Statistical significance was determined using unpaired two‐tailed student's *t* tests.

We investigated the proliferation of HPRT1‐KO cells under 2D and 3D collagen culture conditions (Figure 2A–F). HPRT1 KO slightly reduced the growth of DMS273 cells (Figure 2C) but had no effect on H1048 cells under 2D culture conditions (Figure 2D). In contrast, HPRT1 KO exhibited approximately 70% growth inhibition in both cell lines under 3D collagen culture conditions (Figure 2E,F). These results suggest that the effect of HPRT1 KO on cell proliferation in 3D culture conditions reflects the in vivo outcomes more accurately than 2D culture conditions.

### 3.3 Metabolomic Analysis of HPRT1‐KO Cells in 2D and 3D Culture Models

**FIGURE 3:**
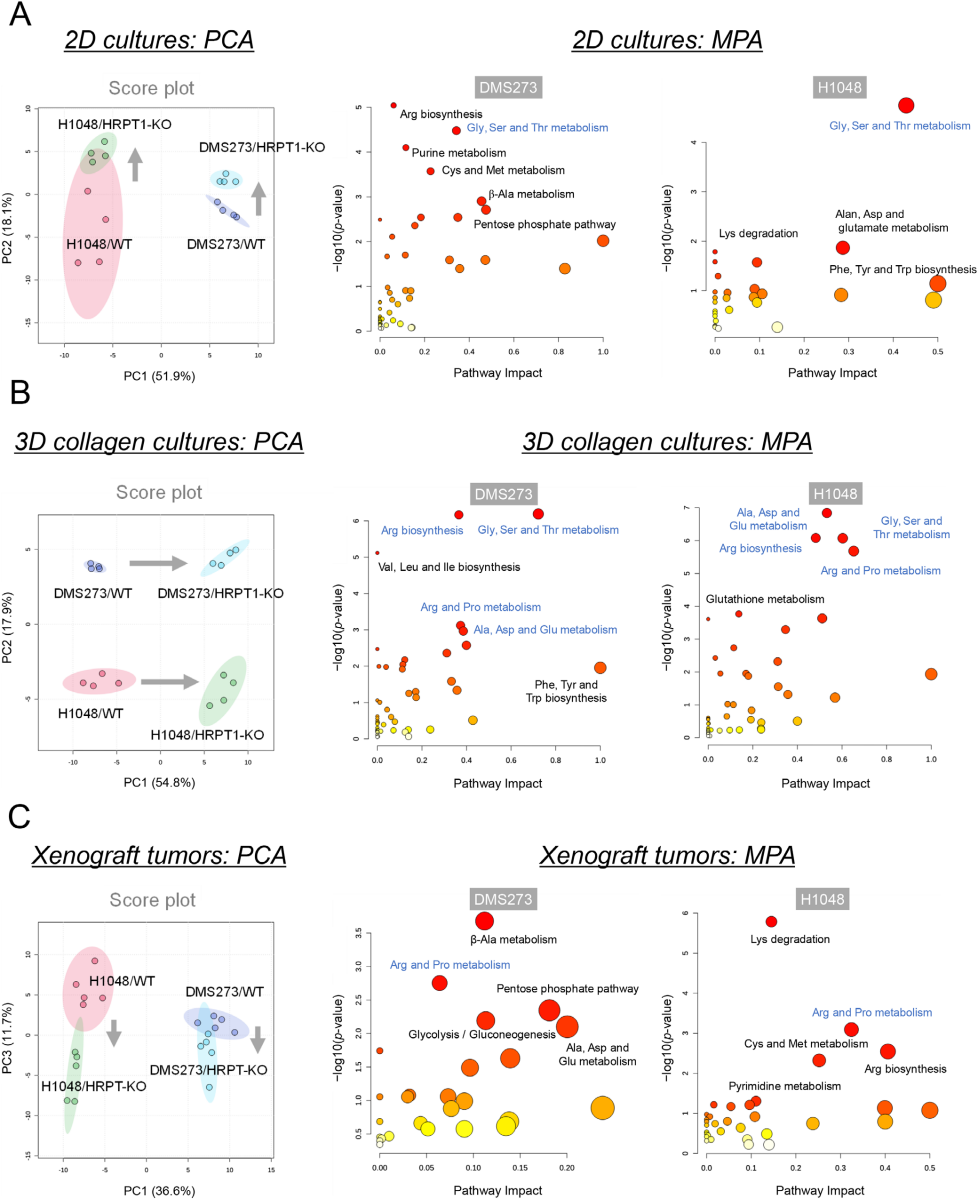
Metabolomic analysis of HPRT1‐KO SCLC cells in 2D/3D culture and in vivo models. (A) Metabolic alterations of DMS273 and H1048 cells with HPRT1 KO in the 2D culture model. Metabolomic analysis was performed using CE‐TOFMS; *N* = 4. PCA (left) and metabolic pathway analysis (MPA, right) of metabolomic data were conducted using Metaboanalyst software. The MPA was evaluated from metabolites differentially expressed in the WT and HPRT1‐KO groups (FDR < 0.05). Blue‐labeled pathways were identified in the MPAs of both DMS273 and H1048 cells. (B) Metabolic alterations of HPRT1‐KO cells in the 3D collagen culture model. (C) Metabolic alterations of HPRT1‐KO cells in the mouse xenograft model.

We investigated the metabolic alterations induced by HPRT1 KO in different culture models (Table S2). First, we conducted metabolomic analysis of DMS273 and H1048 cells lacking *HPRT1* under 2D culture conditions. PCA revealed metabolic differences between cell lines along the first principal component (PC1) axis, whereas HPRT1 KO induced metabolic changes along the second principal component (PC2) axis (Figure 3A, left). In DMS273/HPRT1‐KO cells, 29 and 39 metabolites were significantly increased and decreased, respectively (FDR < 0.05) (Figure S1, top). In contrast, seven metabolites were significantly increased, whereas 49 metabolites were decreased in H1048/HPRT1‐KO cells (Figure S1, bottom). The MPA of significantly altered metabolites showed changes in multiple amino acid metabolism pathways and purine metabolism in DMS273/HPRT1‐KO cells (Figure 3A, right). Similarly, H1048/HPRT1‐KO cells exhibited changes in amino acid metabolism pathways, with Gly, Ser, and Thr metabolism commonly affected in both cell lines.

We performed metabolomic analysis of HPRT1‐KO cells in 3D culture models, utilizing both 3D collagen and honeycomb systems (Table S2). HPRT1‐KO‐induced metabolic changes were observed along the PC1 axis in the 3D collagen culture model, suggesting a greater impact compared to HPRT1‐KO under 2D culture conditions (Figure 3B, left). In DMS273/HPRT1‐KO cells, 66 and 15 metabolites were significantly increased and decreased, respectively (FDR < 0.05) (Figure S2, top). Eighty‐three metabolites significantly increased whereas two decreased in H1048/HPRT1‐KO cells (Figure S2, bottom). Consistent with 2D culture models, MPA revealed prominent changes in amino acid metabolic pathways. Pathways such as Arg biosynthesis, Gly, Ser, and Thr metabolism, Arg and Pro metabolism, and Ala, Asp, and Glu metabolism were commonly altered in both cell lines (Figure 3B, right). The metabolic pathways affected by HPRT1‐KO were more consistent between the two cell lines than those in the 2D culture model.

Metabolomic analysis of HPRT1‐KO cells cultured in the 3D honeycomb model (Table S2, Figure S3) revealed distinct results compared to the 3D collagen culture model. Beyond amino acid metabolic pathways, changes in nicotinate and nicotinamide metabolism, pantothenate and CoA biosynthesis, and the TCA cycle were observed in DMS273/HPRT1‐KO cells.

### 3.4 Metabolomic Analysis of HPRT1‐KO Cells In Vivo

We performed metabolomic analysis of tumor tissues obtained from xenograft mouse models to investigate the metabolic alterations induced by HPRT1 KO in vivo (Table S3) [15]. PCA of metabolomic data revealed metabolic changes along the PC3 axis in HPRT1‐KO tumors (Figure 3C, left). In DMS273/HPRT1‐KO tumors, 24 metabolites were significantly increased and 7 were decreased (FDR < 0.05) (Figure S4, top). Moreover, 17 metabolites were increased and 20 were decreased in H1048/HPRT1‐KO tumors (Figure S4, bottom). MPA of these significantly altered metabolites revealed multiple amino acid metabolic pathways in both cell lines. Additionally, changes in the pentose phosphate pathway and glycolysis were observed in DMS273/HPRT1‐KO tumors, suggesting alterations in energy metabolism (Figure 3C, right).

**FIGURE 4:**
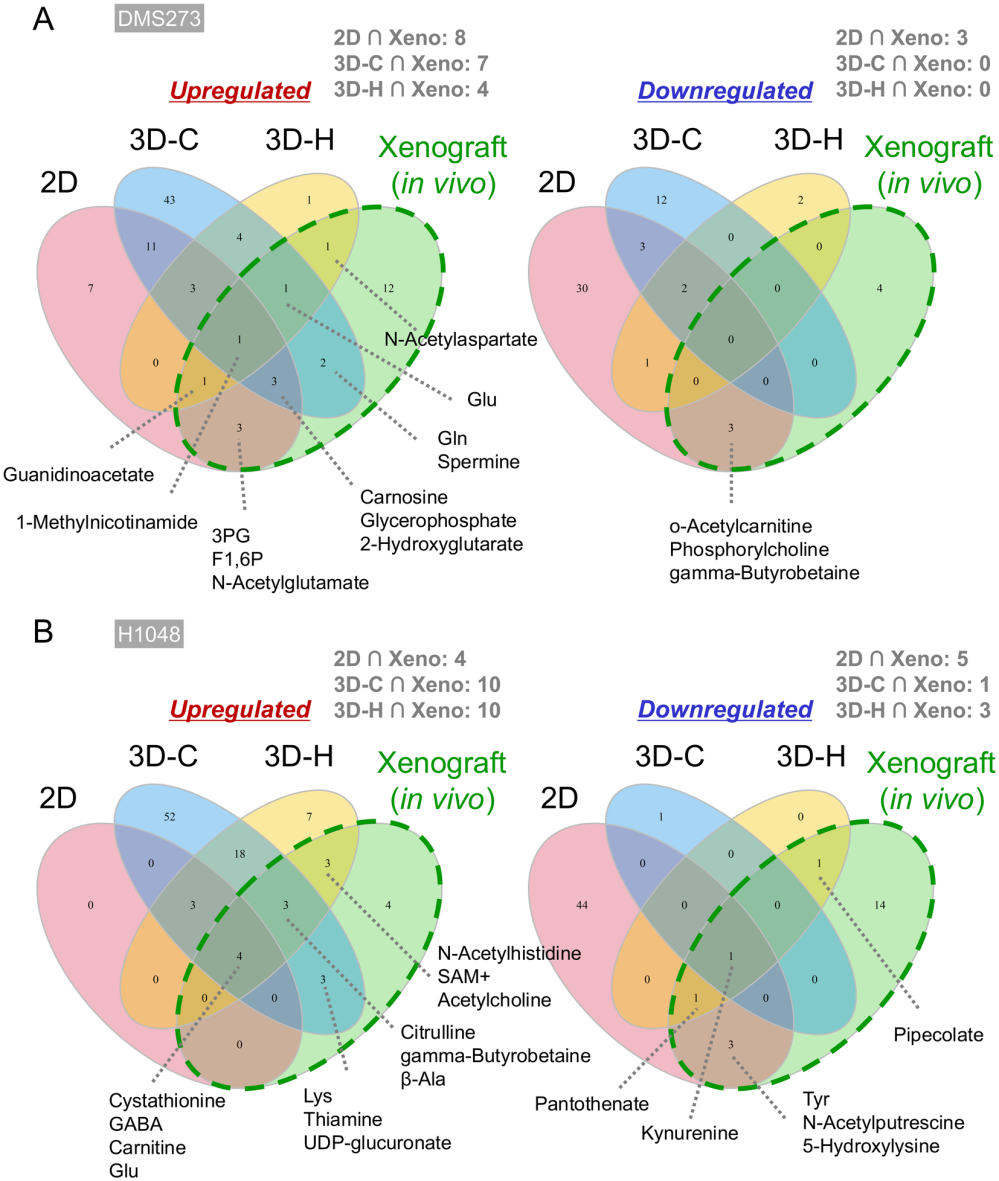
Similarities in metabolites altered by HPRT1 KO in each in vitro model in the in vivo model. (A, B) Venn diagrams showing metabolites differentially expressed by HPRT1‐KO in 2D, 3D collagen (3D‐C), 3D honeycomb (3D‐H), and mouse xenograft models (FDR < 0.05). DMS237 cells (A). H1048 cells (B). The labeled metabolites were commonly identified in both in vitro and in vivo models. Left: Metabolites whose expression was upregulated by HPRT1 KO. Right: Metabolites whose expression was downregulated by HPRT1 KO.

We further identified overlapping metabolites across the different culture models and the xenograft tumor model to compare the metabolic changes induced by HPRT1 KO (FDR < 0.05) in vitro and in vivo (Figure 4). For DMS273 cells, the overlaps were as follows: 2D culture model (2D) ∩ xenograft tumor model (Xeno), eleven metabolites (upregulated: eight, downregulated: three); 3D collagen culture model (3D‐C) ∩ Xeno, seven metabolites (upregulated: seven, downregulated: zero); and 3D honeycomb culture model (3D‐H) ∩ Xeno, four metabolites (upregulated: four, downregulated: zero) (Figure 4A). The expression of 1‐methylnicotinamide was consistently upregulated by HPRT1 KO across all conditions. For H1048 cells, the overlaps were: 2D ∩ Xeno, nine metabolites (upregulated: four, downregulated: five); 3D‐C ∩ Xeno, eleven metabolites (upregulated: ten, downregulated: one); and 3D‐H ∩ Xeno, thirteen metabolites (upregulated: ten, downregulated: three). The expression of cystathionine, GABA, carnitine, and Glu was consistently upregulated, whereas that of kynurenine was consistently downregulated across all conditions (Figure 4B).

**FIGURE 5:**
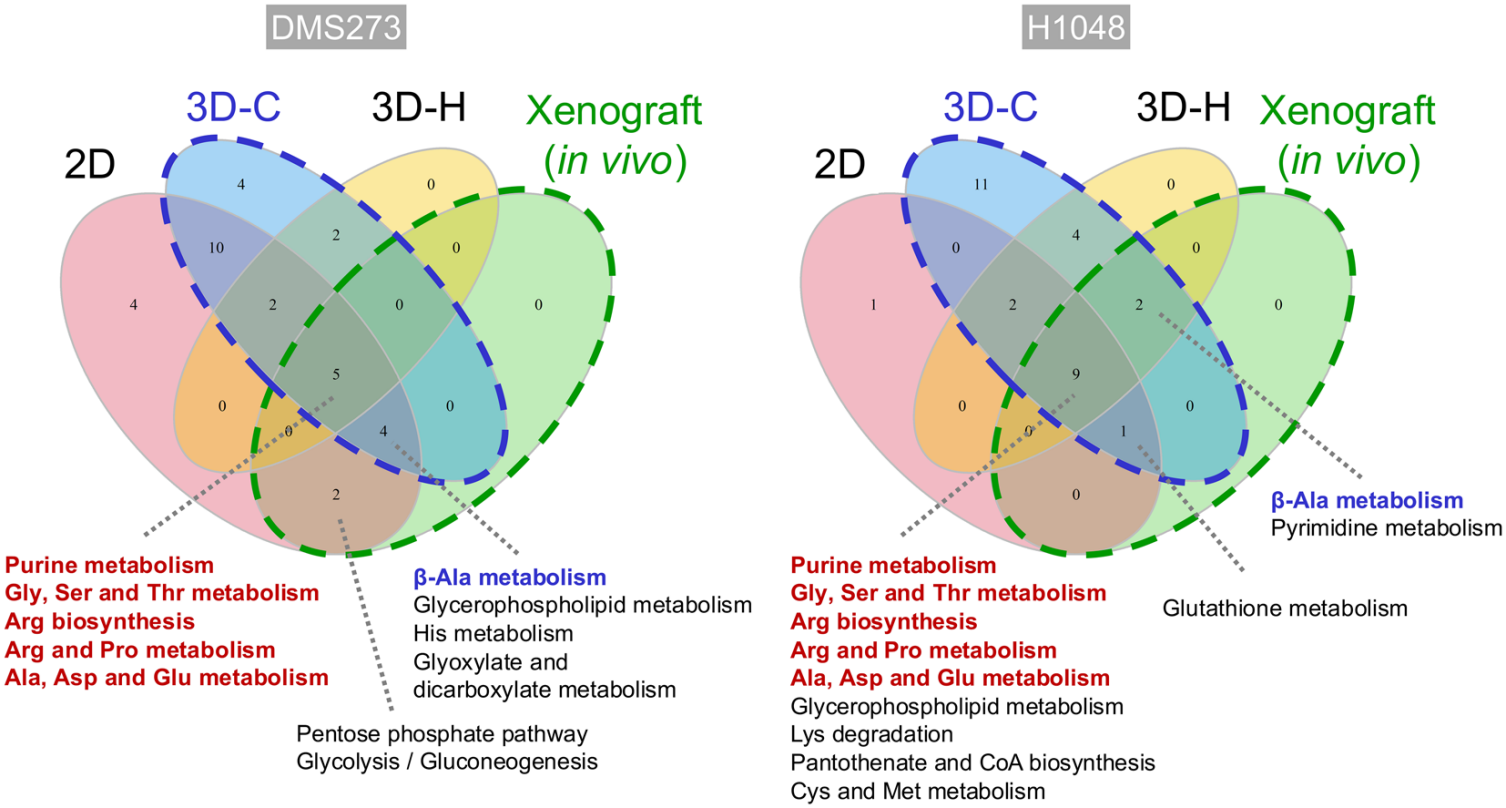
Metabolic pathways altered by HPRT1 KO in each in vitro model in common with the in vivo model. Venn diagrams showing metabolic pathways identified in the MPA in Figure 3. The labeled metabolites were commonly identified pathways both in each in vitro model and in vivo.

The metabolite profiles of DMS273 and H1048 cells varied (Figure 4), thereby complicating the evaluation of the commonality of metabolic changes induced by HPRT1 KO. Therefore, we evaluated pathways common to each model, and the MPA results were compared (Figure 5). Purine metabolism and multiple amino acid metabolic pathways were commonly altered in both cell lines across all models. In particular, β‐Ala metabolism was consistently identified in both cell lines in the 3D collagen culture and xenograft tumor models. β‐Ala metabolism changes were specific to in vivo and 3D culture models in H1048 cells.

### 3.5 HPRT1‐KO‐Induced Production of the Antitumor Metabolite Carnosine in 3D Cultures

**FIGURE 6:**
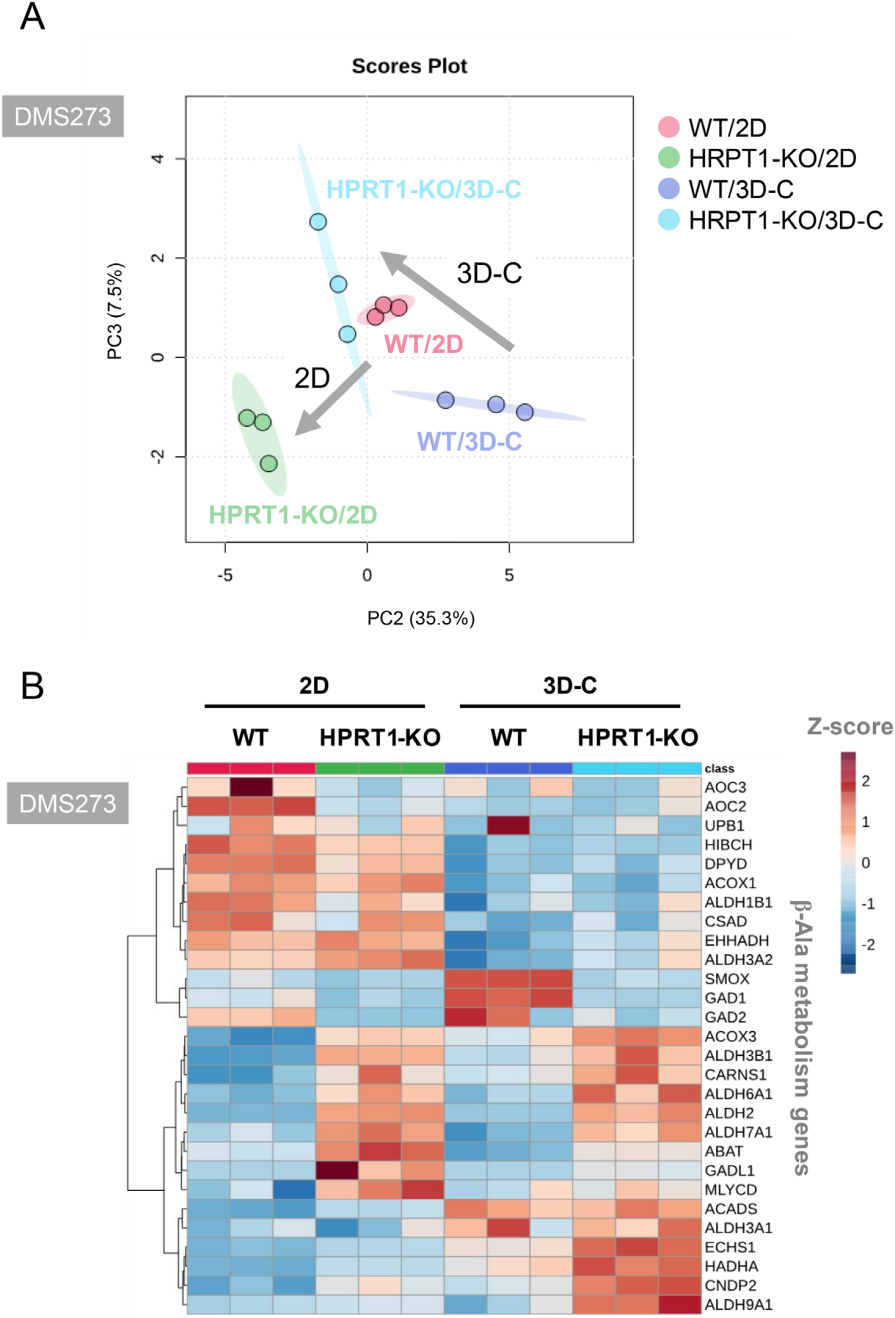
Expression of β‐Ala metabolism genes in HPRT1‐KO DMS273 cells under 2D and 3D culture conditions. (A) PCA of β‐Ala metabolism genes in DMS273 cells under 2D and 3D collagen (3D‐C) culture conditions. Gene expression was determined using RNA‐seq analysis; *N* = 3. (B) Expression of β‐Ala metabolism genes in DMS273 under 2D and 3D‐C culture conditions. The heatmap shows the *Z*‐score of relative gene expression for each sample; *N* = 3.

**FIGURE 7:**
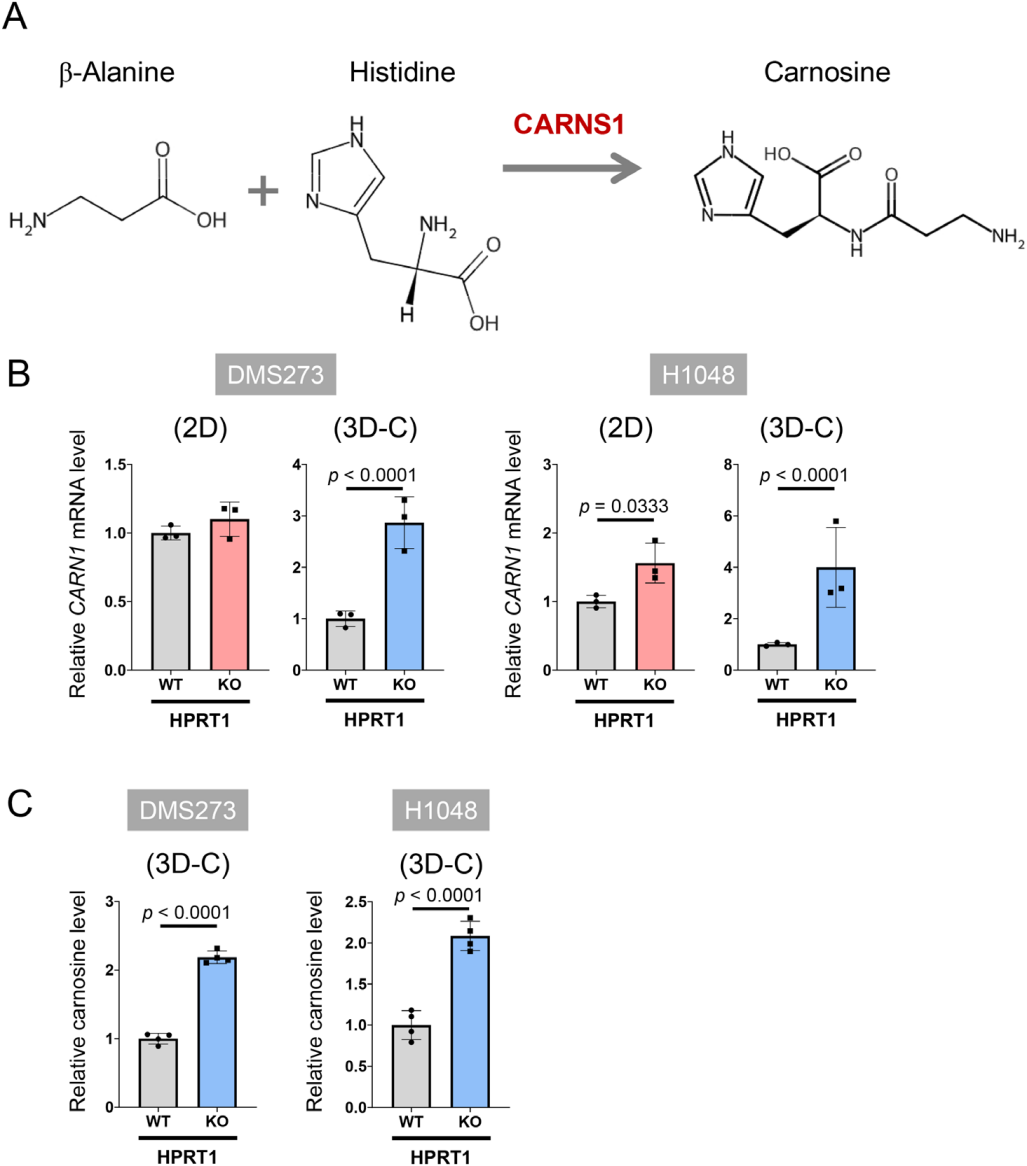
Upregulation of *CARNS1* mRNA and carnosine expression by HPRT1‐KO in SCLC cells under 3D culture conditions. (A) A schematic diagram showing the enzymatic reaction of CARNS1. (B) Expression of *CARNS1* mRNA in DMS273 and H1048 cells with HPRT1 KO in the 2D and 3D collagen culture models. mRNA expression levels of *CARNS1* were assessed using real‐time PCR; *N* = 3. Data are expressed as the mean ± SD. Statistical significance was determined using an unpaired two‐tailed student's *t* test. (C) Levels of carnosine in DMS273 and H1048 cells with HPRT1 KO in the 3D collagen culture models. Carnosine levels were measured with CE‐TOFMS; *N* = 3.

Next, we examined the expression of 26 genes related to the β‐Ala metabolic pathway in HPRT1‐KO cells in the 2D and 3D collagen models. PCA plots of gene expression along the PC2 and PC3 (Figure 6A) axes and the heatmap (Figure 6B) revealed distinct expression profiles between 2D and 3D collagen culture models. Additionally, HPRT1 KO induced different gene expression patterns between these models (Figure 6A,B). Notably, HPRT1 KO significantly increased the expression of *CARNS1* (Figure 7B), which converts β‐Ala and His to carnosine (Figure 7A), and intracellular carnosine levels in both cell lines in the 3D collagen culture model (Figure 7C).

### 3.6 *CARNS1* Expression Improves Prognosis in Lung Cancer and Negatively Correlates With HPRT1 Expression

**FIGURE 8:**
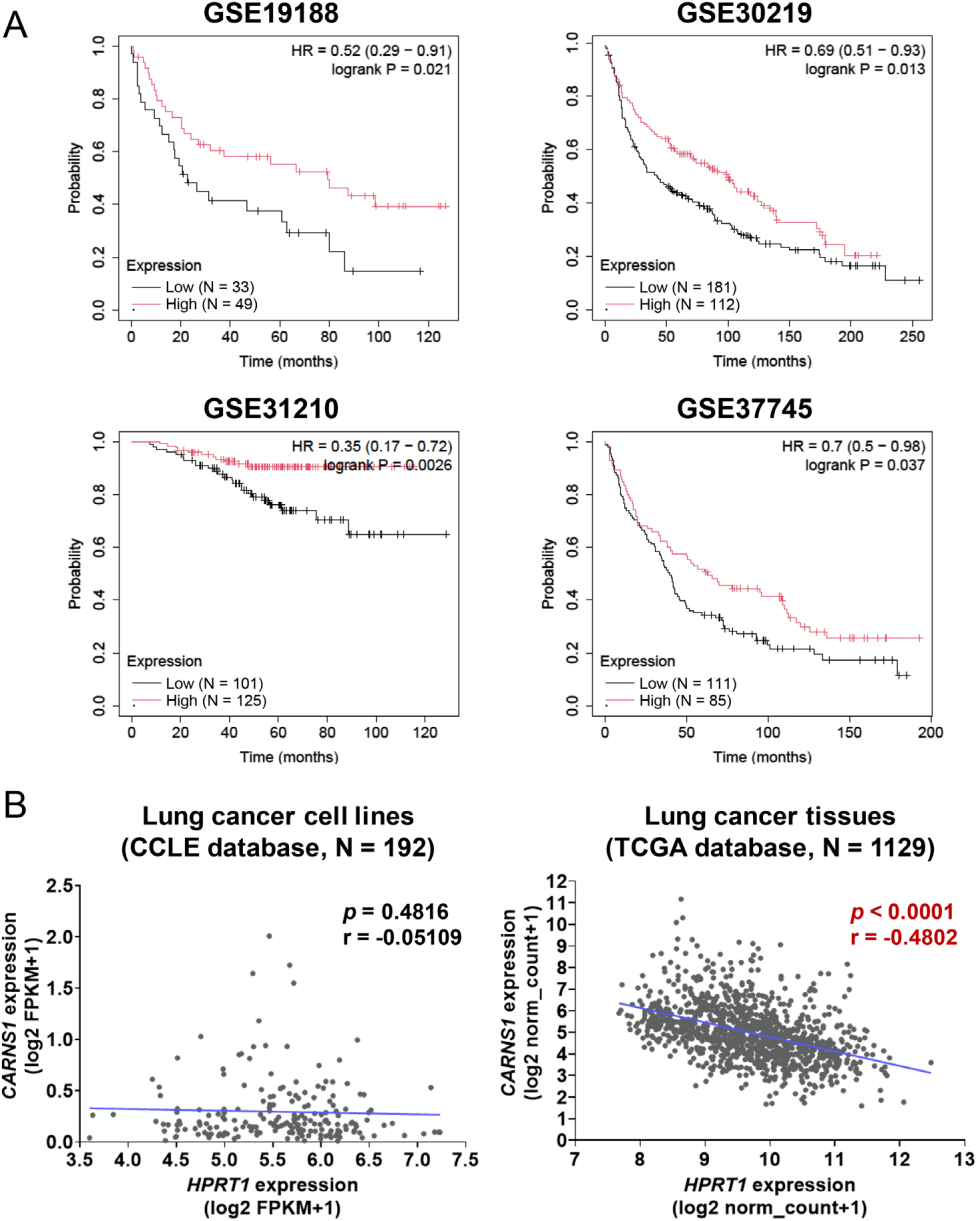
Clinical impact of CARNS1 expression in lung cancer. (A) Kaplan–Meier curves of low and high CARNS1 expression in public datasets of patients with lung cancer (GSE19188, GSE30219, GSE31210, and GSE37745). (B) Correlations between CARNS1 and HPRT1 mRNA expression in the lung cancer cell lines (left, *N* = 192) and tissues (right, *N* = 1129) in the CCLE and TCGA datasets, respectively. Regression is indicated with a blue line. Correlation was analyzed using the Peason's correlation.

Carnosine inhibits tumor growth in vivo [27, 28], and increased *CARNS1* expression indicates enhanced carnosine production and antitumor effects. To explore the role of *CARNS1* expression in cancer malignancy, we used public databases to analyze its association with lung cancer prognosis (Figure 8A). Because the public data specific to SCLC were not available, this analysis was conducted using data that included all types of lung cancer. Patients with high *CARNS1* expression had significantly better prognoses in four independent clinical datasets. We also investigated the relationship between *HPRT1* and *CARNS1* expression using CCLE (lung cancer cell lines cultured in 2D) and TCGA (clinical lung cancer tissues) databases (Figure 8B). Although the CCLE data showed no correlation, TCGA data revealed a significant negative correlation between *HPRT1* and *CARNS1* expression. These findings suggest that the relationship between *CARNS1* and *HPRT1* is tissue‐specific.

**FIGURE 9:**
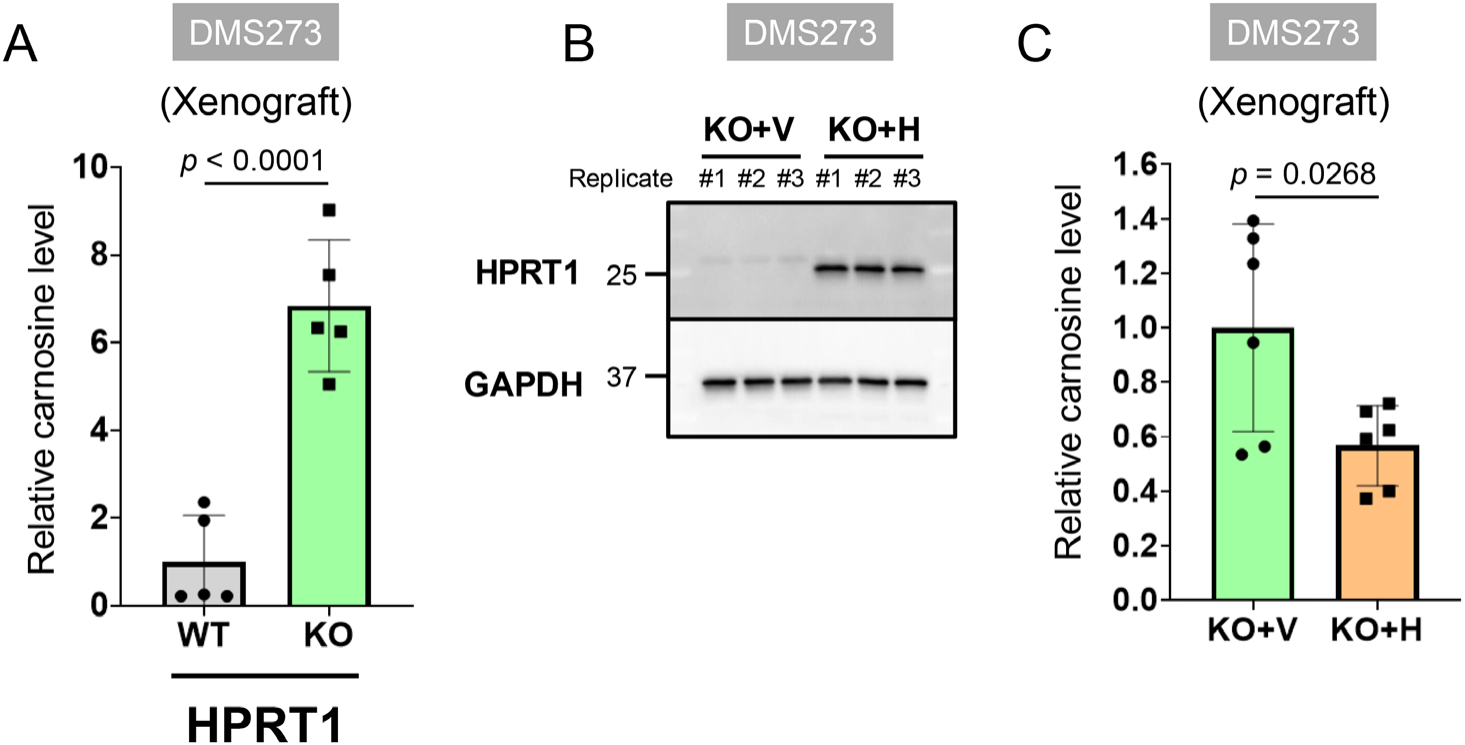
Levels of carnosine in HPRT1‐KO tumors in vivo. (A) Carnosine levels in xenograft tumors from HPRT1‐KO DMS273cells. Carnosine levels were measured using CE‐TOFMS; *N* = 5. Data are expressed as the mean ± SD. Statistical significance was determined using unpaired two‐tailed student's *t* tests. (B) Expression of HPRT1 in the HPRT1‐KO DMS273 cells transfected with the control vector (KO + V) and *HPRT1* cDNA (KO + H) in 2D cultures. Protein expression levels were measured using western blotting. GAPDH was used as the loading control; *N* = 3. (C) Rescue effect of HPRT1 on carnosine levels in tumors from HPRT1‐KO DMS273 cells transfected with the control vector (KO + V) and *HPRT1* cDNA (KO + H); *N* = 6.

We further analyzed carnosine levels in xenograft tumors derived from DMS273/HPRT1‐KO cells using CE‐TOFMS to determine whether HPRT1 KO enhances carnosine production in vivo. Carnosine levels were found to be higher in HPRT1‐KO tumors than in HPRT1‐WT tumors (Figure 9A). Additionally, the reintroduction of *HPRT1* into HPRT1‐KO cells reduced carnosine levels (Figure 9B,C). These findings demonstrate that HPRT1 negatively regulates carnosine production in vivo.

## 4 Discussion

This study highlights the importance of 3D culture systems for elucidating context‐dependent metabolic regulation and evaluating therapeutic targets in cancer, using HPRT1‐KO cells with a dysfunctional purine salvage pathway as a model. The reduction in cell proliferation in 3D culture and xenograft models indicates the importance of 3D systems. In addition, differences in metabolic profiles between the 2D and 3D culture models following HPRT1 KO demonstrate the impact of the extracellular environment on tumor metabolism. Furthermore, we observed that HPRT1 suppresses carnosine production within the β‐Ala metabolic pathway in 3D collagen cultures and in vivo, but not in a 2D culture. This finding indicates 3D‐context metabolic regulation by HPRT1.

The differential effects of HPRT1 KO on cell proliferation in 2D versus 3D and in vivo models are potentially attributable to differences in nutrient availability, oxygen levels, and cell–cell interactions. These environmental factors may also influence the mechanisms by which HPRT1 regulates β‐Ala metabolism. In particular, metabolomic analysis of cells cultured in 2D and 3D (Figure 1B) revealed substantial differences in the levels of various amino acids, suggesting that these changes may affect HPRT1‐mediated control of β‐Ala metabolism. However, the molecular mechanisms by which HPRT1 regulates gene expression and enzyme activity in the β‐Ala metabolic pathway, particularly those of CARNS1, remain largely unexplored and require further clarification.

Additionally, across all three models—2D, 3D, and mouse xenografts—HPRT1 KO consistently induced alterations in purine metabolism as well as in amino acid metabolic pathways such as Gly and Arg metabolism (Figure 5). These pathways are either directly regulated by HPRT1 enzymatic activity or closely linked to its metabolic function, making the observed metabolic changes biologically plausible. While these pathways generally contribute to the synthesis of DNA or RNA, the context‐dependent differences in their functional roles are unclear and require further investigation.

The experimental models used in this study were selected based on their biological relevance to tumor physiology. The 2D culture allows for high‐throughput analysis under controlled conditions but lacks physiological complexity. The 3D culture partially recapitulates in vivo conditions, including cell–cell and cell–extracellular matrix interactions, oxygen gradients, and spatial architecture. In contrast, mouse xenograft models reflect systemic signaling and host interactions, making them indispensable for evaluating clinical relevance. By integrating these three models, we elucidated context‐dependent metabolic phenotypes and assessed their translational relevance. Carnosine is an endogenous dipeptide composed of β‐Ala and L‐His, and its endogenous concentration is up to 20 mM in the human body. This dipeptide is mainly localized in the brain and skeletal muscle [29]. It possesses anti‐aging [30], cerebroprotective [31], anti‐oxidant [32], and anti‐inflammatory [33] properties, along with Parkinson's disease inhibitory effects [34]. In addition, the anticancer effects of carnosine have been demonstrated in various cancer types, such as breast cancer [28], ovarian cancer [35], gastric cancer [36], colorectal cancer [37], prostate cancer [38] and leukemia [39], indicating its potential as an anticancer agent [27]. For instance, Zn‐carnosine prevented dysphagia in patients with breast cancer treated with adjuvant radiation therapy in a phase III clinical trial [40]. The molecular functions of carnosine in cancer cells include cell cycle arrest, apoptosis, proteolysis, and dysfunction of energy metabolism [27, 41]; however, its direct target remains unclear.

Comprehensive analysis of clinical lung cancer datasets further supported the therapeutic potential of carnosine and its association with HPRT1‐mediated carnosine regulation. High CARNS1 expression was correlated with improved survival in patients with lung cancer (Figure 8A). Moreover, HPRT1 and CARNS1 expression levels were negatively correlated in clinical tumor samples, whereas no such correlation was observed in 2D‐cultured cell lines (Figure 8B). This tissue‐specific relationship highlights the importance of investigating metabolic regulation within the tumor microenvironment. In addition, gene expression analysis of the β‐Ala metabolic pathway (Figure 6) indicated that the metabolic impact of HPRT1 KO may vary depending on the cellular growth environment. These findings suggest that in vitro–in vivo metabolic discrepancies could significantly influence the development of diagnostic strategies and therapeutic targets. Our results indicate that 3D culture systems can reflect physiological metabolic interactions involving HPRT1 and carnosine, supporting their utility in identifying clinically relevant metabolic targets, stratifying patients, and predicting therapeutic efficacy.

Limitations and future directions. While CE‐TOFMS enabled sensitive and high‐resolution detection of polar metabolites, it was less effective for hydrophobic or neutral molecules. Therefore, additional analytical strategies will be required to ensure comprehensive metabolic coverage. Furthermore, the expression of carnosine and CARNS1 was assessed at a single time point; therefore, further time‐course studies are necessary to elucidate how HPRT1 regulates CARNS1 expression under 3D culture conditions. Although Figure 9 shows that carnosine levels are inversely correlated with HPRT1 expression in vivo upon KO and reconstitution, the direct impact of carnosine on tumor growth has not yet been evaluated. In addition, owing to the limited availability of clinical SCLC specimens, direct comparisons of metabolic profiles between cells cultured in 2D or 3D models, mouse xenografted tumors, and human tumors remain incomplete.

Although 3D culture systems replicate spatial structure and cell–cell interactions better than 2D models, they lack essential noncancerous cell components of the tumor microenvironment, such as immune cells and fibroblasts. These stromal cells play crucial roles in regulating tumor metabolism through paracrine signaling, nutrient exchange, and immunometabolic crosstalk. For example, cancer‐associated fibroblasts (CAFs) support cancer cell proliferation by secreting lactate and amino acids, thereby rewiring tumor cell metabolism [42]. Similarly, tumor‐infiltrating immune cells such as T cells and macrophages can alter nutrient availability and produce immunosuppressive metabolites such as kynurenine, influencing both tumor growth and immune evasion [43]. Therefore, future studies should aim to incorporate coculture or organotypic models that include multiple stromal cell types to more accurately recapitulate the metabolic complexity of the tumor microenvironment.

In summary, our findings demonstrate that HPRT1 regulates carnosine metabolism under 3D conditions, influencing tumor growth. 3D culture systems are effective tools for uncovering metabolic regulations that remain undetectable in 2D cultures. This study underscores the potential of 3D culture models for fundamental cancer metabolism research and for guiding precision oncology through improved model fidelity.

## Author Contributions

**Sho Tabata:** conceptualization (lead), data curation (lead), formal analysis (lead), funding acquisition (equal), investigation (lead), methodology (equal), project administration (lead), visualization (lead), writing – original draft (lead). **Ichiro Fujimoto:** methodology (equal), resources (equal), supervision (equal), writing – review and editing (equal). **Tomoyoshi Soga:** investigation (equal), resources (equal), writing – review and editing (equal). **Hideki Makinoshima:** funding acquisition (equal), project administration (equal), resources (equal), supervision (equal), writing – review and editing (equal).

## Funding

This study was supported by JSPS KAKENHI, Grant Number 22K07150 (S.T.); JSPS KAKENHI, Grant Number 24K10418 (H.M.); Japan Health Research Promotion Bureau (JH) Research Fund, Grant Number JH2024‐B‐07 (H.M.); and research funds from the Yamagata Prefecture Government, Japan, and City of Tsuruoka, Japan (S.T., H.M., and T.S.).

## Ethics Statement

Animal experiments were approved by the Animal Care Committee of the National Cancer Center (Approval number: #17502 and #21502).

## Conflicts of Interest

T.S. is an Associate Editor of Cancer Science. I.F. is an employee of Koken Co. Ltd. The other authors have no conflicts of interest.

## Supporting information


**Figure S1:** Metabolic alterations of HPRT1‐KO SCLC cells in the 2D culture model.
**Figure S2:** Metabolic alterations of HPRT1‐KO SCLC cells in the 3D collagen culture model.
**Figure S3:** Metabolomic analysis of HPRT1‐KO SCLC cells in 3D honeycomb culture.
**Figure S4:** Metabolic alterations of HPRT1‐KO SCLC cells in a mouse xenograft model.

**Table S1:** Primer sequences for qPCR.

**Table S2:** Levels of metabolites in HPRT1‐KO SCLC cells.

**Table S3:** Levels of metabolites in tumors from HPRT1‐KO H1048 cells.

## Data Availability

Metabolomic data are included in Tables [Link], [Link]. All other data are available upon reasonable request.
