# Supplementary material for: Context‐Specific Metabolic Alterations in HPRT1 Knockout Cells Within a 3D Culture System
Source: Cancer Med. 2025 Dec 9;14(23):e71452. doi: 10.1002/cam4.71452 (PMC12688478; doi:10.1002/cam4.71452)
Supplement: Supplementary file 1 — Figure S1: Metabolic alterations of HPRT1‐KO SCLC cells in the 2D culture model. Figure S2: Metabolic alterations of HPRT1‐KO SCLC cells in the 3D collagen culture model. Figure S3: Metabolomic analysis of HPRT1‐KO SCLC cells in 3D honeycomb culture. Figure S4: Metabolic alterations of HPRT1‐KO SCLC cells in a mouse xenograft model. [file CAM4-14-e71452-s003.pdf]

Fig. S1

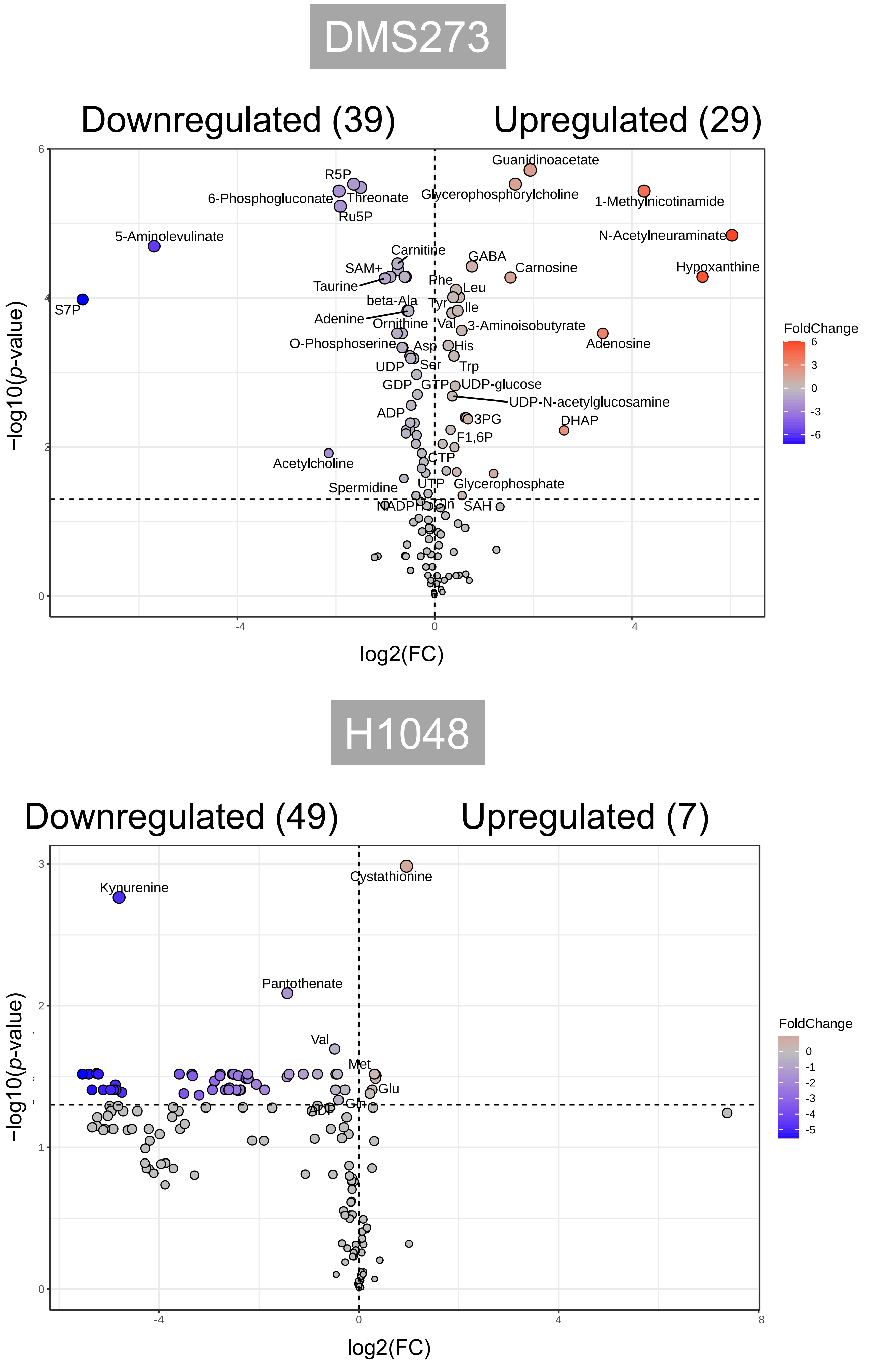

Fig. S1. Metabolic alterations of HPRT1-KO SCLC cells in the 2D culture model. Volcano plots showing differences in metabolite levels in DMS273 (top) and H1048 (bottom) cells with HPRT1 KO (FDR < 0.05). Metabolomic analysis was performed using CE-TOFMS (N = 4).

Fig. S2

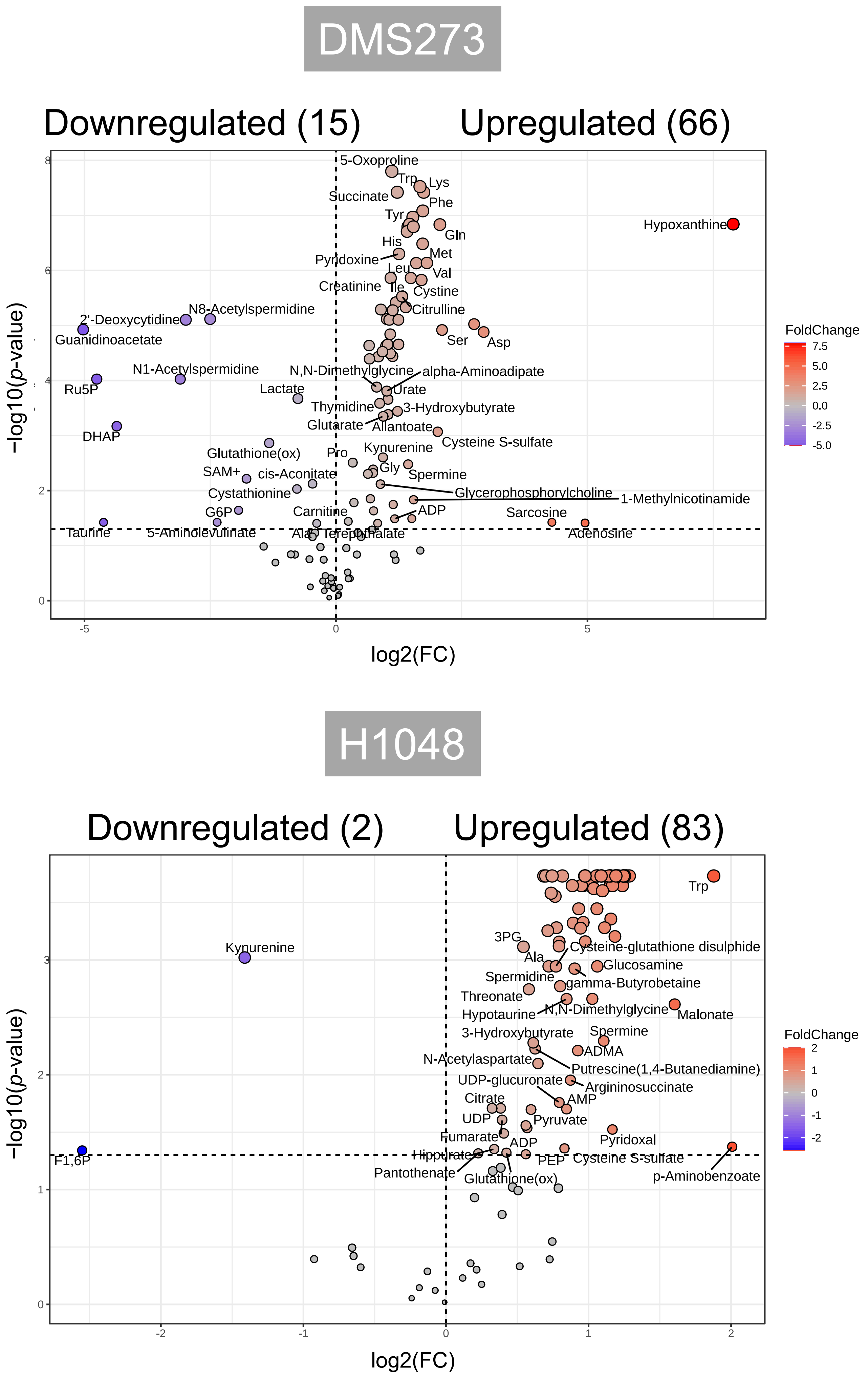

Fig. S2. Metabolic alterations of HPRT1-KO SCLC cells in the 3D collagen culture model. Volcano plots showing differences in metabolite levels in DMS273 (top) and H1048 (bottom) cells with HPRT1 KO (FDR < 0.05). Metabolomic analysis was performed using CE-TOFMS (N = 4).

Fig. S3

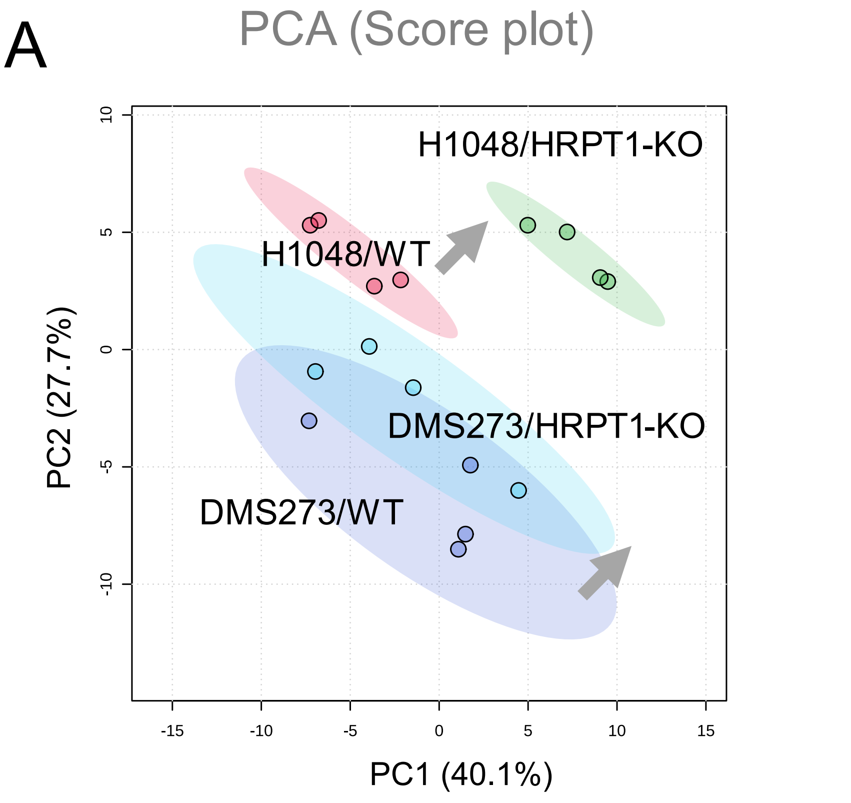

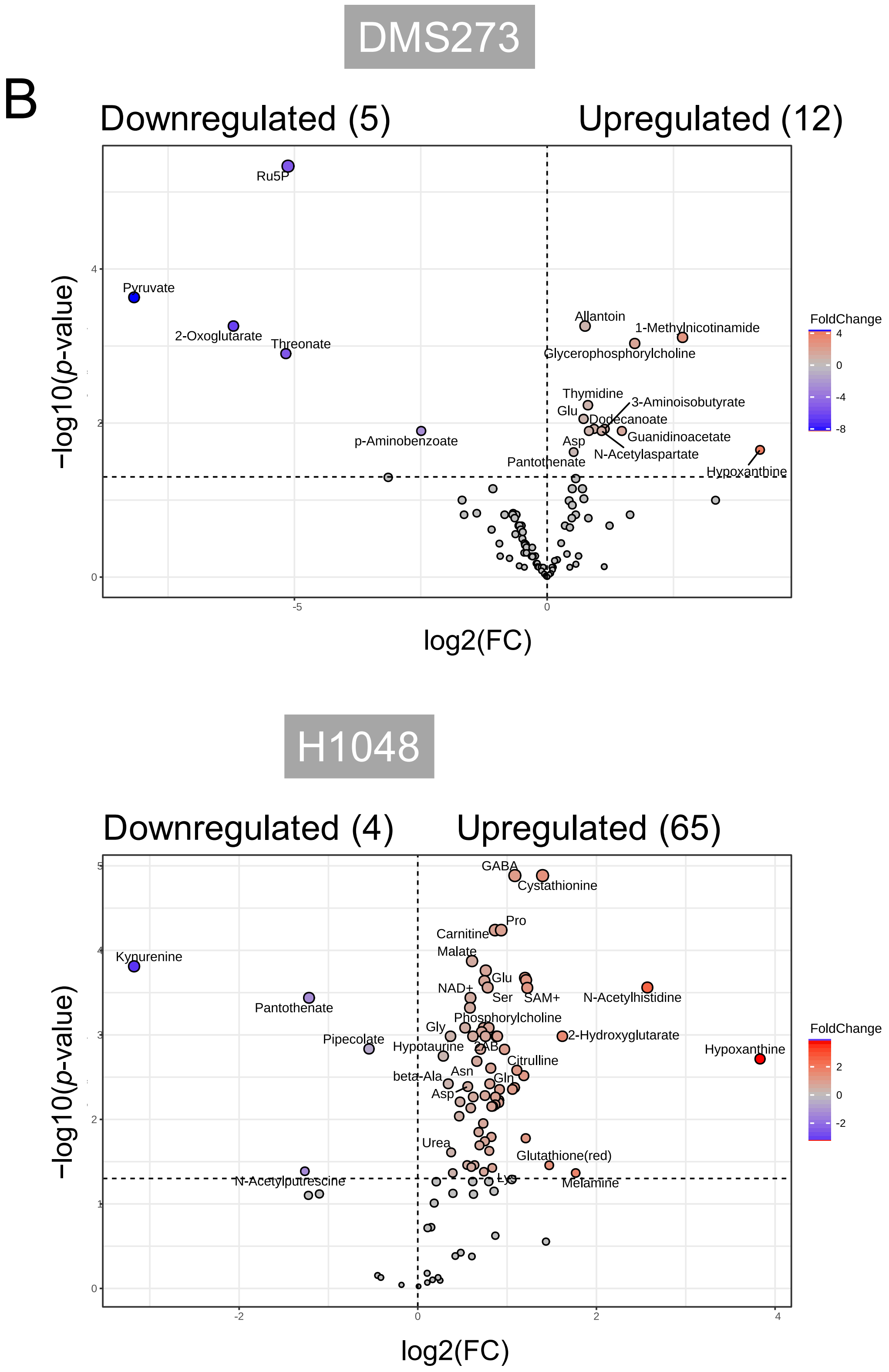

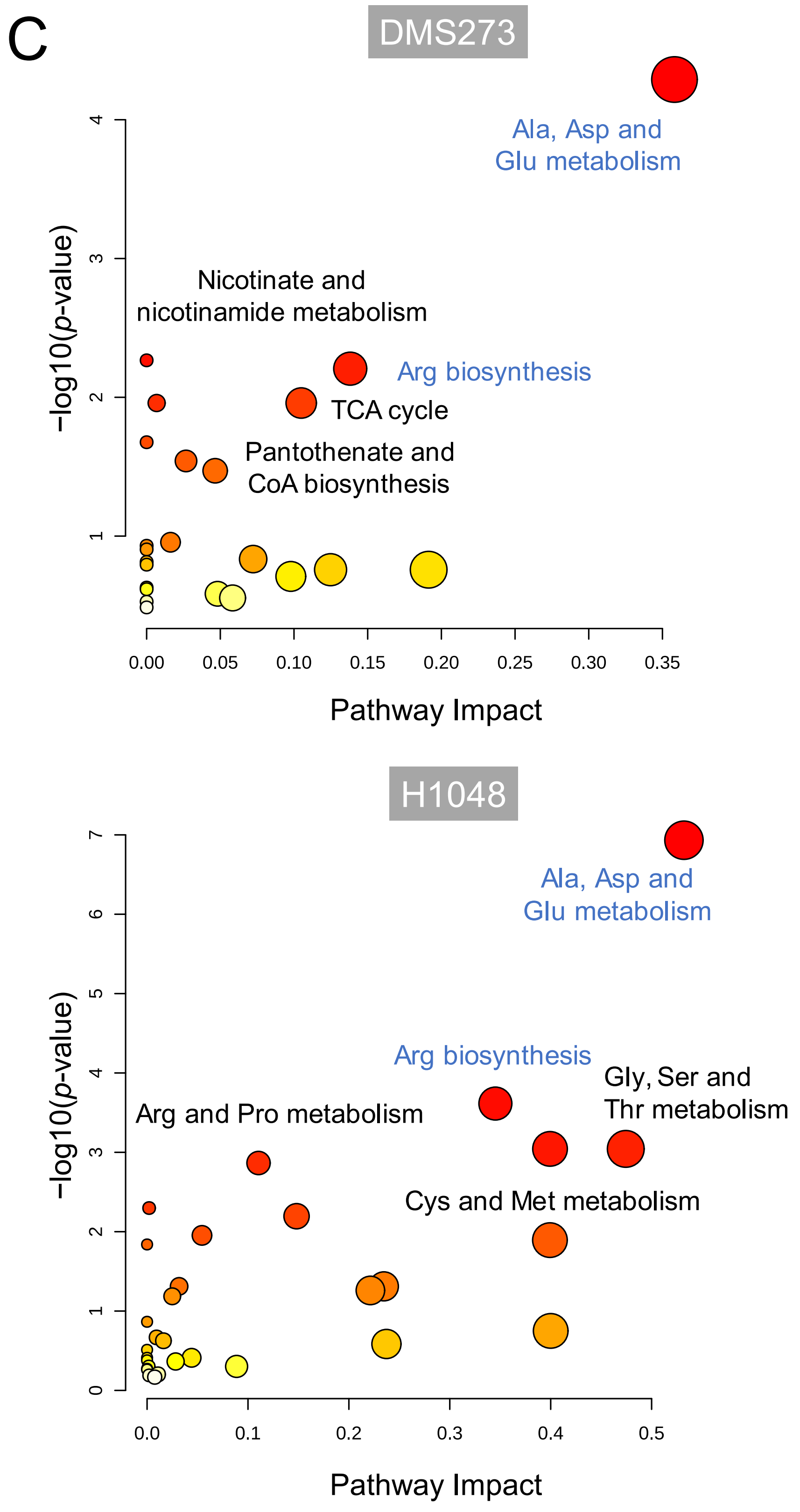

Fig. S3. Metabolomic analysis of HPRT1-KO SCLC cells in 3D honeycomb culture. (A) Principal component analysis (PCA) of metabolites in DMS273 and H1048 cells with HPRT1 KO in 3D honeycomb culture model. Metabolomic analysis was performed using CE-TOFMS; N = 4. (B) Volcano plots showing differences in metabolite levels in DMS273 (top) and H1048 (bottom) cells with HPRT1 KO (FDR < 0.05). (C) Metabolic pathway analysis of the differentially expressed metabolites (FDR < 0.05) in the WT vs. HPRT1-KO groups.

Fig. S4

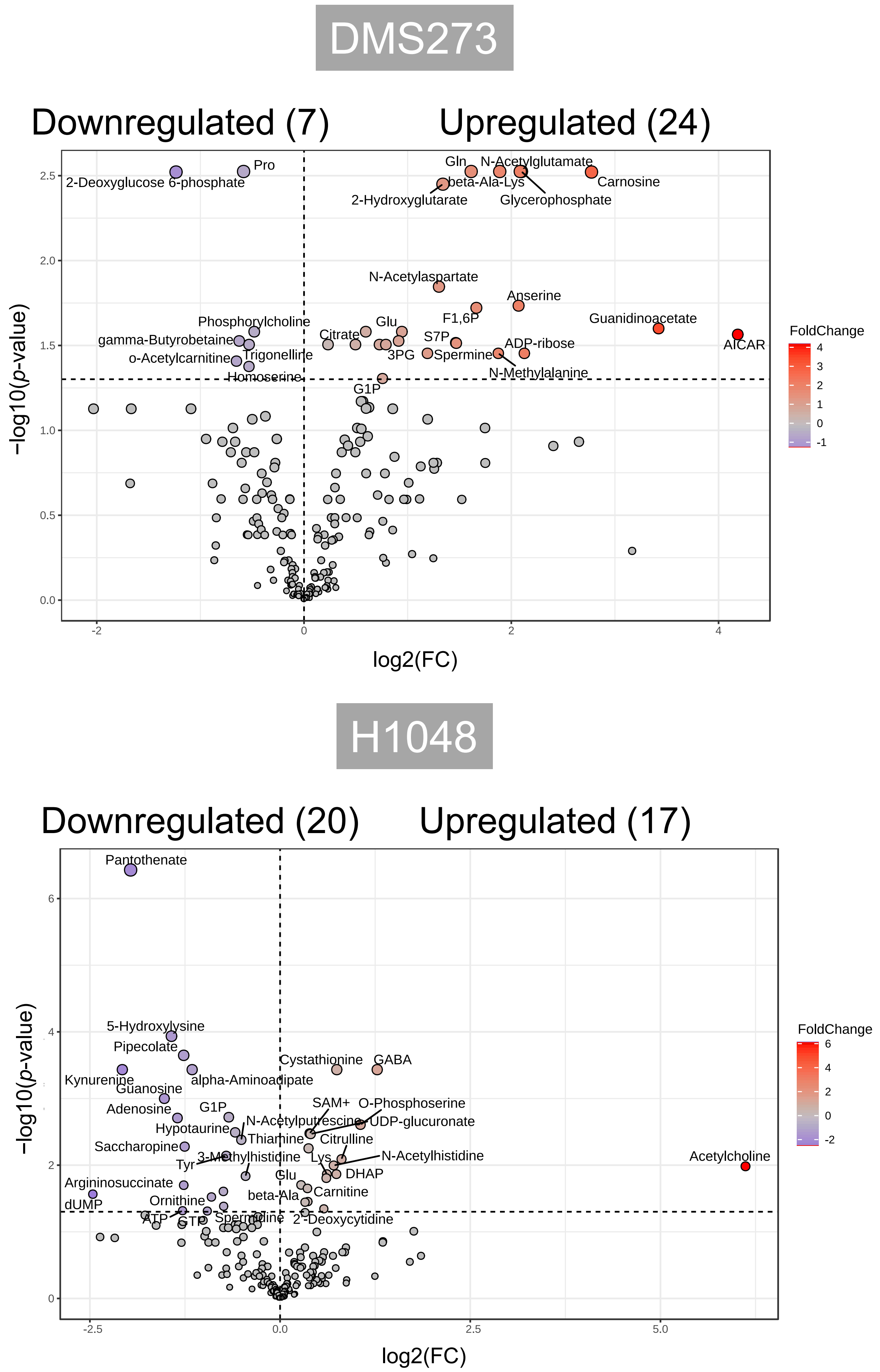

Fig. S4. Metabolic alterations of HPRT1-KO SCLC cells in a mouse xenograft model. Volcano plots showing differences in metabolite levels in DMS273 (top) and H1048 (bottom) cells with HPRT1 KO (FDR < 0.05). Metabolomic analysis was performed using CE-TOFMS; N = 4.
